# Functional Groups Response to Water Deficit in Mediterranean Ecosystems

**DOI:** 10.3390/plants12071471

**Published:** 2023-03-27

**Authors:** Helena Castro, Maria Celeste Dias, José Paulo Sousa, Helena Freitas

**Affiliations:** Centre for Functional Ecology, TERRA Associate Laboratory, Department of Life Sciences, University of Coimbra, Calçada Martim de Freitas, 3000-456 Coimbra, Portugal

**Keywords:** *Cistus*, drought, legumes, forbs, grasses, shrubs

## Abstract

Enhanced drought, more frequent rainfall events and increased inter-annual variability of precipitation are the main trends of climate expected for the Mediterranean. Drought is one of the most important stressors for plants and significantly impacts plant communities causing changes in plant composition and species dominance. Through an experiment under controlled conditions, we assessed the response of Mediterranean species from different functional groups (annual grass, annual forb, annual legume, and perennial shrub) to moderate and severe water deficit. Changes in plant traits (leaf dry matter), biomass and physiology (water status, photosynthesis, pigments, and carbohydrate) were evaluated. The studied species differed in their response to water deficit. *Ornithopus compressus*, the legume, showed the strongest response, particularly under severe conditions, decreasing leaf relative water content (RWC), pigments and carbohydrates. The grass, *Agrostis pourreti* and the forb, *Tolpis barbata*, maintained RWC, indicating a higher ability to cope with water deficit. Finally, the shrub, *Cistus salviifolius*, had the lowest response to stress, showing a higher ability to cope with water deficit. Despite different responses, plant biomass was negatively affected by severe water deficit in all species. These data provide background for predicting plant diversity and species composition of Mediterranean grasslands and Montado under climate change conditions.

## 1. Introduction

Predictions for the Mediterranean region point to increased inter-annual variability of precipitation and an increase in extreme climatic events such as heatwaves and severe droughts as main and prevalent trends [1]. Drought is one of the most important stressors for plants and significantly impacts plant communities causing changes in plant composition and species dominance, as well as in plant traits and secondary metabolites, with the potential to affect decomposition and nutrient cycling through changes in both litter quality and quantity [2,3,4]. Rainfall, particularly during spring, affected plant diversity and species composition in Mediterranean grasslands, with legumes being more affected than grasses and forbs [2,5]. Co-occurring species may therefore differ in their response to water deficit. Although the reduction in biomass production is a generalized response of plants to water stress, the patterns of biomass allocation and the physiological responses vary with species and functional groups [6,7]. It is, therefore, necessary to understand the response to climate change for as many different growth forms as possible so that it is possible to evaluate the vulnerability of highly diverse ecosystems, such as the Mediterranean ecosystems. In this study, we explore the response of four species common in open Montado areas [2,8], belonging to different functional groups, to different levels of water deficit.

Plants possess a wide range of mechanisms to cope with environmental stress, including stress caused by water deficit, that can be grouped into three significant strategies [6,9,10,11]: (1) Escape, achieved by modifying phenology and shortening their life cycle.; (2) Avoidance of water deficits, which can be achieved through strategies that increase water uptake and/or decrease water loss; (3) Tolerance to water deficit, in which plants can survive under decreased water availability. Mechanisms involved in increasing water acquisition include increased water uptake, generally by the increased depth and density of roots, whereas mechanisms involved in decreasing water loss include, for example, stomatal closure, reduction in leaf area and leaf rolling [9,11]. The mechanisms involved in dehydration tolerance include turgor maintenance (e.g., accumulation of solutes) and desiccation tolerance, which can be achieved using biochemical and morphological strategies to protect cells from injury [9,11]. These strategies may lead to various physiological and biochemical changes [11].

Drought stress usually causes a significant reduction in water potential and stomatal conductance for CO_2_ due to stomatal closure. The suboptimal CO_2_ assimilation rates often result in light absorption exceeding the demand for photosynthesis [12], and the excess energy may lead to the production of reactive oxygen species (ROS), which, if not counterbalanced by antioxidant defences, will lead to photo-oxidation [13]. It can also affect the light depending on the phase of photosynthesis, resulting in lower photosynthetic efficiency (maximum and effective efficiency of photosystem II), decreased growth and biomass production, and leaf senescence [13,14]. Plants have several mechanisms to deal with excess light energy. The non-photochemical quenching (NPQ), which is associated with the carotenoids involved in the xanthophyll cycle, helps in the dissipation of the excess energy from light-harvesting when light energy absorption exceeds the capacity for light utilization [15,16]. Under water deficit conditions, many plants increase carotenoids’ content to cope with oxidative stress and/or decrease chlorophylls’ content, thereby decreasing the amount of light absorbed and avoiding oxidative stress (e.g., *Olea europaea* [17], *Arbutus unedo* [18], *Rosmarinus officinalis* [19], and *Stipa tenacissima* [20]).

Osmotic adjustment is a way to maintain cell turgor and is a process in which cellular metabolism is altered to increase cellular solute concentrations, allowing the maintenance of cellular water content via diffusion [21]. The solutes that accumulate in the cells include sugars, proline, and quaternary ammonium compounds (e.g., glycine-betaine). In addition, osmolytes may enhance drought tolerance by stabilizing proteins and reducing oxidative stress that often arises when plants are subjected to water deficit [22,23,24,25]. Increases in total soluble sugars have been reported for some species (e.g., *Triticum aestivum* [26] and *Sorghum bicolor* [21]).

We evaluated the response to water availability of four Mediterranean species belonging to different functional groups (annual grass, annual forb, annual legume, and perennial shrub). Our main hypothesis is that functional groups respond differently to water deficit, with the legume species being the most affected and the perennial shrub being the least affected. We set up an experiment under controlled conditions to test our hypothesis, including three water treatments (control, moderate water deficit and severe water deficit). In addition, we assessed the response of physiologic variables such as plants’ water status, photosynthesis, photosynthetic pigments (chlorophylls and carotenoids) and carbohydrates (soluble sugars and starch), of leaf traits such as leaf dry matter content (LDMC) and biomass-related traits.

## 2. Results

**Figure 1:**
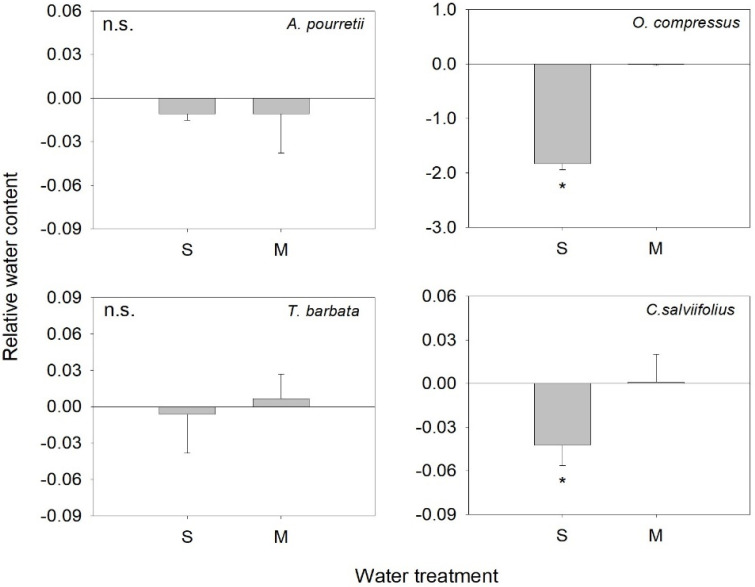
Response ratio (mean values + SE) for the response ratio of the relative water content of *Agrostis pourretii*, *Ornithopus compressus*, *Tolpis barbata* and *Cistus salviiflolius* under severe water deficit (S) and moderate water deficit (M). The presence of * indicates that the response ration differed significantly from zero. n.s., not significant.

**Figure 2:**
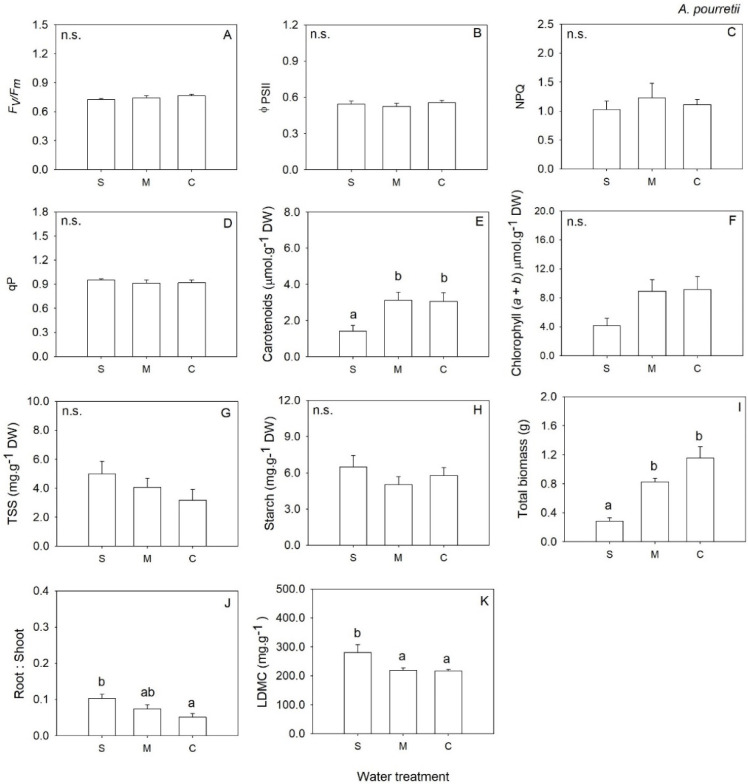
Mean values (+SE) of maximum quantum efficiency of photosystem II (**A**), the effective quantum efficiency of PSII (**B**), non-photochemical quenching (**C**), photochemical quenching (**D**), carotenoids content (**E**), chlorophylls (*a* + *b*) (**F**), total soluble sugars (**G**), starch content (**H**), total biomass (**I**), root to shoot biomass ratio (**J**) and Leaf Dry Matter Content (**K**) in *Agrostis pourretti* grown under severe water deficit (S), moderate water deficit (M) and control (C) conditions. Significant differences at *p* < 0.05 among treatments are indicated by different letters. n.s., not significant.

The response of RWC to water deficit treatment varied, and we only observed a significant response for severe water deficit and for *O. compressus* and *C. salviifolius*, which showed a decrease in leaf water (Figure 1). Similarly, for the remaining traits, the response to water deficit treatment varied with the functional group and trait analyzed. In annual grass *A. pourretii*, biomass was significantly lower in severe water deficit (S) compared to moderate water deficit (M) and control (C) (Table 1, Figure 2I). But there was an increase in the root: shoot ratio with decreasing water availability (Table 1, Figure 2J). Carotenoids were significantly lower, and chlorophylls were lower by marginal differences (*p* = 0.057) in S plants compared to M and C (Table 1, Figure 2E,F). LDMC was significantly higher in S compared to M and C groups (Table 1, Figure 2K). Plants under S conditions produced a significantly lower number of flowers when compared to M and C plants (Table 2). The remaining traits were not affected by water availability (Table 1, Figure 2B–D,G,H,J).

**Figure 3:**
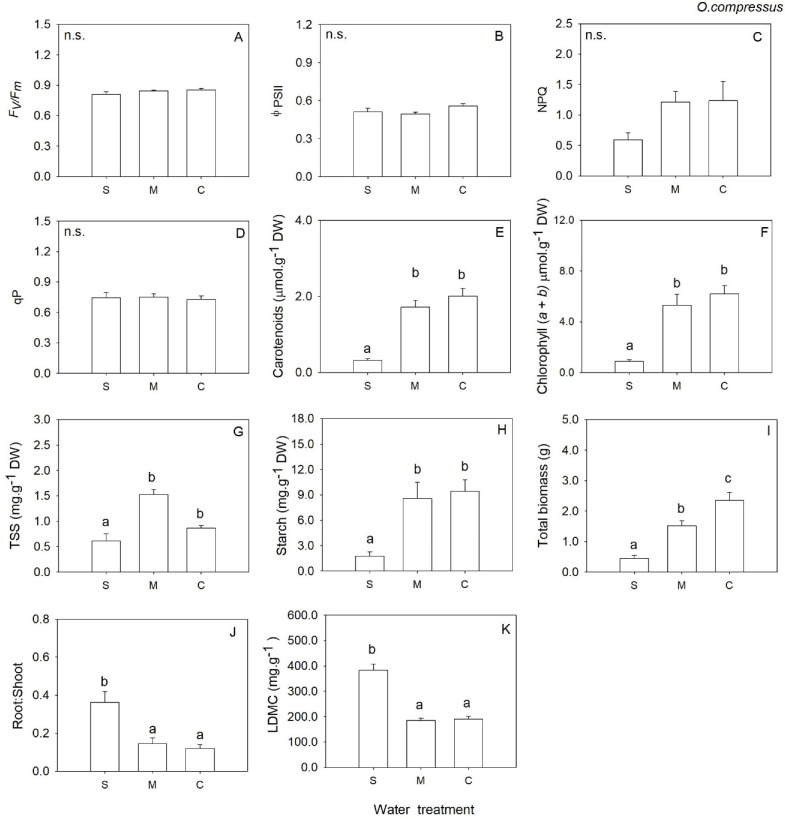
Mean values (+SE) of maximum quantum efficiency of photosystem II (**A**), the effective quantum efficiency of PSII (**B**), non-photochemical quenching (**C**), photochemical quenching (**D**), carotenoids content (**E**), chlorophylls (*a* + *b*) (**F**), total soluble sugars (**G**), starch content (**H**), total biomass (**I**), root to shoot biomass ratio (**J**) and Leaf Dry Matter Content (**K**) *Ornithopus compressus* grown under severe water deficit (S), moderate water deficit (M) and control (C) conditions. Significant differences at *p* < 0.05 among treatments are indicated by different letters. n.s., not significant.

In annual legume *O. compressus*, biomass decreased significantly with decreasing water availability (Table 1, Figure 3I), but the plants under severe water deficit (S) invested proportionally more in root biomass than in shoot biomass compared to M and C (root: shoot ratio significantly higher in S than M and C; Table 1, Figure 3J). *O. compressus* plants under severe water deficit showed significantly lower levels of carotenoids, chlorophylls and starch when compared to plants under moderate water deficit and control (Table 1, Figure 3E,F,H). TSS were higher in plants under moderate water deficit when compared S and C (Table 1, Figure 3G). LDMC was higher in severe water deficit when compared to M and C (Table 1, Figure 3K). Additionally, *O. compressus* plants under severe water deficit did not produce any flowers, while plants under moderate water deficit and control produced, on average, 16.83 ± 2.51 and 5.76 ± 2.35, respectively (Table 2).

**Figure 4:**
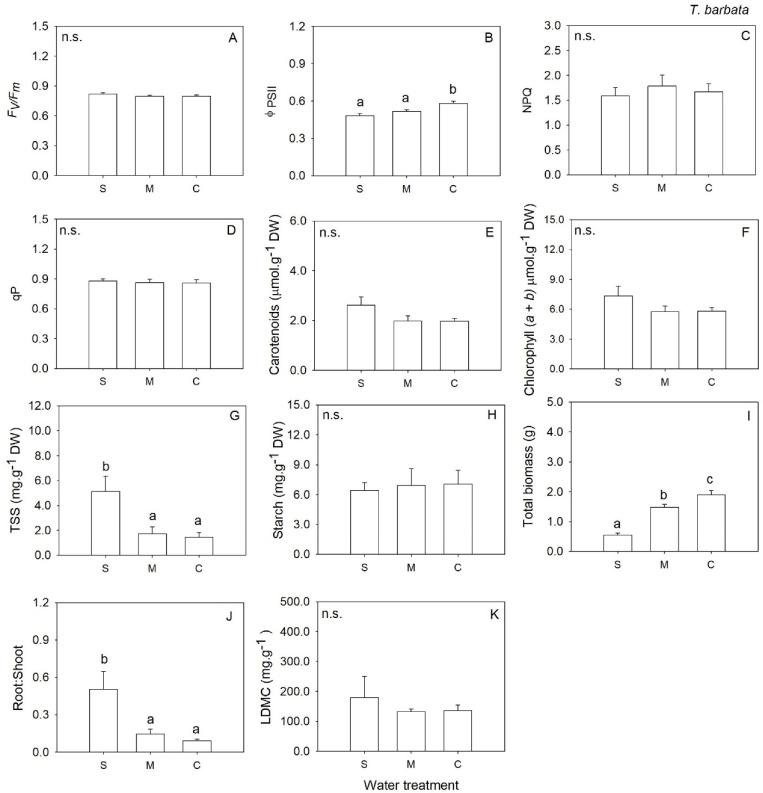
Mean values (+SE) of maximum quantum efficiency of photosystem II (**A**), the effective quantum efficiency of PSII (**B**), non-photochemical quenching (**C**), photochemical quenching (**D**), carotenoids content (**E**), chlorophylls (*a* + *b*) (**F**), total soluble sugars (**G**), starch content (**H**), total biomass (**I**), root to shoot biomass ratio (**J**) and Leaf Dry Matter Content (**K**) *Tolpis barbata* grown under severe water deficit (S), moderate water deficit (M) and control (C) conditions. Significant differences at *p* < 0.05 among treatments are indicated by different letters. n.s., not significant.

In annual forb *T. barbata* biomass was significantly lower in severe water deficit when compared to M and C (Table 1, Figure 4I). But the plants under severe water deficit invested proportionally more in root biomass than in shoot biomass compared to M and C (root: shoot ratio significantly higher in S than M and C; Table 1, Figure 4J). Φ_PSII_ was lower, and TSS was higher in severe water deficit than in M and C (Table 1, Figure 4B,G). Plants under severe water deficit produced a significantly lower number of flowers when compared to M and C (Table 2). The remaining traits were not affected by water availability (Table 1, Figure 4A,C–F,H,L).

**Figure 5:**
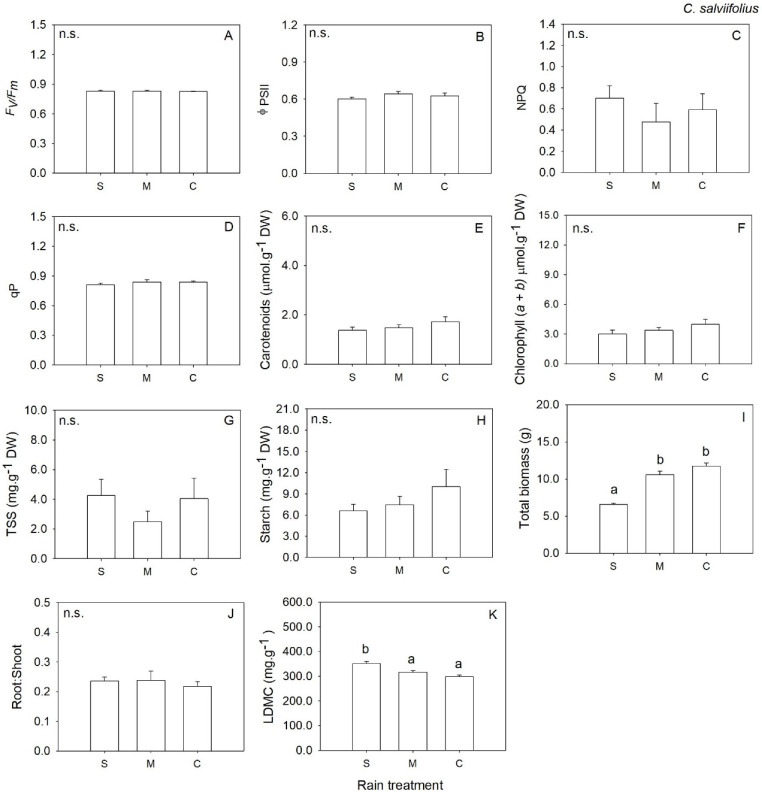
Mean values (+SE) of maximum quantum efficiency of photosystem II (**A**), the effective quantum efficiency of PSII (**B**), non-photochemical quenching (**C**), photochemical quenching (**D**), carotenoids content (**E**), chlorophylls (*a* + *b*) (**F**), total soluble sugars (**G**), starch content (**H**), total biomass (**I**), root to shoot biomass ratio (**J**) and Leaf Dry Matter Content (**K**) *Cistus salviifolius* grown under severe water deficit (S), moderate water deficit (M) and control (C) conditions. Significant differences at *p* < 0.05 among treatments are indicated by different letters. n.s., not significant.

**Figure 6:**
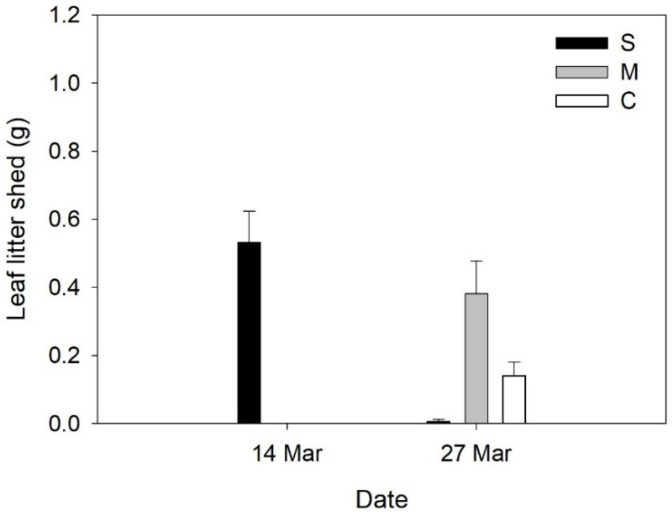
Leaf litter (mean values +SE) shed by *Cistus salviifolius* on different dates after the start of water deficit treatments on 3 March 2017 for plants under severe water deficit (S), moderate water deficit (M) and control (C).

In perennial shrub, *C. salviifolius* biomass was significantly lower in severe water deficit when compared to M and C (Table 1, Figure 5I). LDMC was significantly higher in severe water deficit than M and C (Table 1, Figure 5K). Photosynthesis-related traits and root: shoot ratio were not significantly affected by water availability (Table 1, Figure 5A–H,J). Plants in severe water deficits shed leaves earlier than in M and C pots. Plants under severe water deficit produced 88.0% of the total leaf litter shed during the experiment two weeks after the beginning of water deficit treatment, while leaf litter shed in moderate water deficit and control occurred two weeks later (Table 1, Figure 6). 

**Figure 7:**
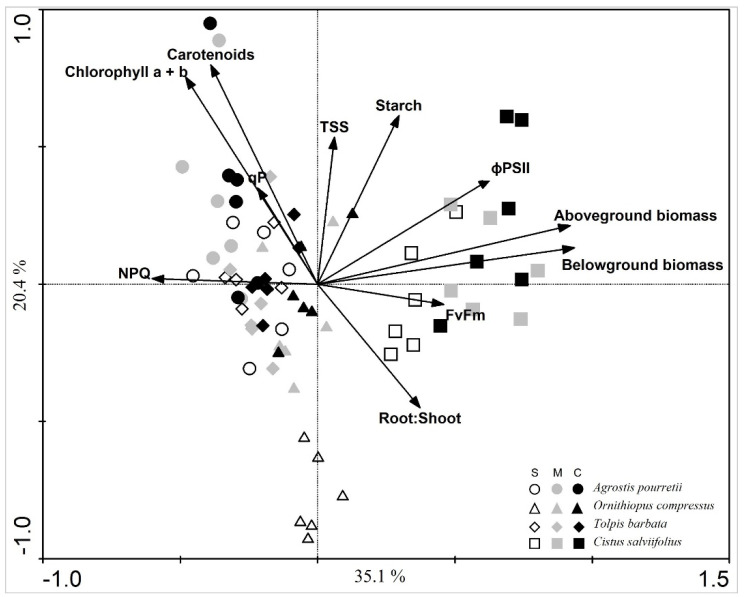
Scatterplots of principal components analysis of physiologic and morphologic variables of plants of each functional group under severe water deficit, moderate water deficit and control.

The PCA separates shrub species *C. salviifolius* from the herbaceous species. The *O. compressus* under severe water deficit are grouped apart from other plants in the PCA, both from other species and from other plants of the same species under moderate water deficit and control (Figure 7). The PCA also shows a strong correlation between chlorophylls and carotenoid content (Figure 7).

## 3. Discussion

Co-occurring species may differ in their response to water deficit, with species and functional groups showing different patterns of biomass allocation and physiological responses [6,7]. Therefore, we hypothesized that the four Mediterranean species used in this study, which belong to different functional groups, would differ in their response to different levels of water deficit. 

The PCA separates the shrub *C. salviifolius* from the herbaceous species. Indeed, this species showed the lowest response to water deficit. We only found a response to severe stress and only for RWC and biomass, which decreased, and LMDC, which increased in response to severe water deficit. Grant et al. [27] and Puglielli et al. [28] also found a reduction in RWC in response to water deficit stress, but stress protective mechanisms, such as increased stomatal aperture control and osmotic adjustment were activated and helped to cope with drought stress. This species has a high physiological plasticity, displaying a great adaptation to summer drought conditions [28]. *Cistus* species are semi-deciduous, i.e., they are characterized by drought-avoiding phenology, displaying two different leaf cohorts: (1) autumn leaves that burst in late autumn/early winter and last until the following spring (May) when they are shed; (2) summer leaves, which burst in spring and are characterized by higher lamina thickness and trichome density, lower epidermal and mesophyll cells, reduced intercellular spaces and stomata located in crypts, compared to autumn leaves [7]. De Dato et al. [7] reported earlier leaf shed in response to water deficit in *C. monspeliensis* and a reduction of photosynthesis and stomata aperture. In our study, *C. salviifolius,* under severe water deficit, shed over 80% of the autumn leaves two weeks after the beginning of the stress treatment and earlier than moderate water deficit and control (Figure 6). Considering the reports from earlier studies with *Cistus* spp., leaf shedding in summer can be seen as a strategy to reduce water loss through transpiration [27] or as the result of chronic photoinhibition possible to detect by a decrease in F_v_/F_m_ [28]. In our study, leaf shed in severe water deficit stress occurred before chlorophyll fluorescence measurements, which may explain the absence of differences in F_v_/F_m_. It implies that the leaves collected for LDMC in severe water deficit were likely summer leaves, possibly thicker [29], and therefore presenting higher LDMC, while for moderate water deficit and control collected leaves were likely to be autumn leaves. Differences in LDMC may thus result from intrinsic differences in summer vs. autumn leaves rather than a response in LDMC to water deficit stress. 

*Agrostis pourretii* was reported to have isohydric behavior, i.e., it can maintain midday water potential relatively stable as environmental conditions change, which may explain a certain resistance of this species to water stress [30] and the maintenance of the RWC. *A. pourretii* showed drought resistance in combination with a water spender mechanisms upon irrigation in a study evaluating the response of understory herbaceous species of a Mediterranean cork oak shrubland to increasing precipitation variability [30]. Consistent with this, in our study, moderate water deficit did not induce, in general, negative responses. However, stronger effects were visible under severe water deficits. For instance, carotenoids’ content and chlorophylls (not significant) decreased, possibly due to damages resulting from oxidative stress or pigment biosynthesis reduction/inhibition [13]. However, chlorophylls’ decrease did not affect the effective efficiency of PSII (Φ_PSII_), suggesting that ATP and NADP availability for the Calvin Cycle was not compromised by water deficit stress [31]. 

In a study evaluating the resilience of Montado herbaceous species to precipitation variability, *T. barbata* was able to maintain photosynthesis and stomatal conductance under water deficit similar to control, indicating that this species has increased water use efficiency and phenotypic adaptation to drought [32]. In the present work, plants of *T. barbata* appear grouped in the PCA without any clear distinction among water treatments. The photosynthesis measurements showed that both water deficit conditions impaired the efficiency of PSII photochemistry (Φ_PSII_), which can limit Calvin Cycle reactions due to less ATP and NADPH availability [32]. Moreover, severe water deficit increased TSS levels suggesting an increased ability to maintain tissue turgor through osmotic protection [22,28,33,34]. On the other hand, water deficit treatments did not affect leaf RWC, indicating a good hydration status. Besides osmoregulation, soluble sugars are also involved in the regulation of reactive oxygen species signaling, as well as in photosynthesis and mitochondrial respiration [35].

Among the species used, the legume *O. compressus* was expected to show the highest sensitivity to water deficit. Legumes have been shown to be more sensitive to altered precipitation patterns (low soil water availability) under Mediterranean conditions [2,32]. Indeed, the plants under severe water deficit are grouped apart from other plants in the PCA, both from other species and from other plants of the same species exposed to moderate water deficit and control. *O. compressus* plants under severe water deficit showed a loss of chlorophylls and carotenoids, a sign of damage induced by oxidative stress or a reduction/inhibition of pigment biosynthesis [35]. Moreover, TSS and starch content decreased, possibly due to re-translocation to root growth since these plants showed a strong investment in root biomass under a severe water deficit. This investment in root growth may also have contributed to water status maintenance (RWC). Higher sensitivity to water deficit (decrease of water potential, net CO_2_ assimilation rate, stomatal conductance and water use efficiency) was reported by Jongen et al. [30] for *Ornithopus sativus*. 

**Figure A1:**
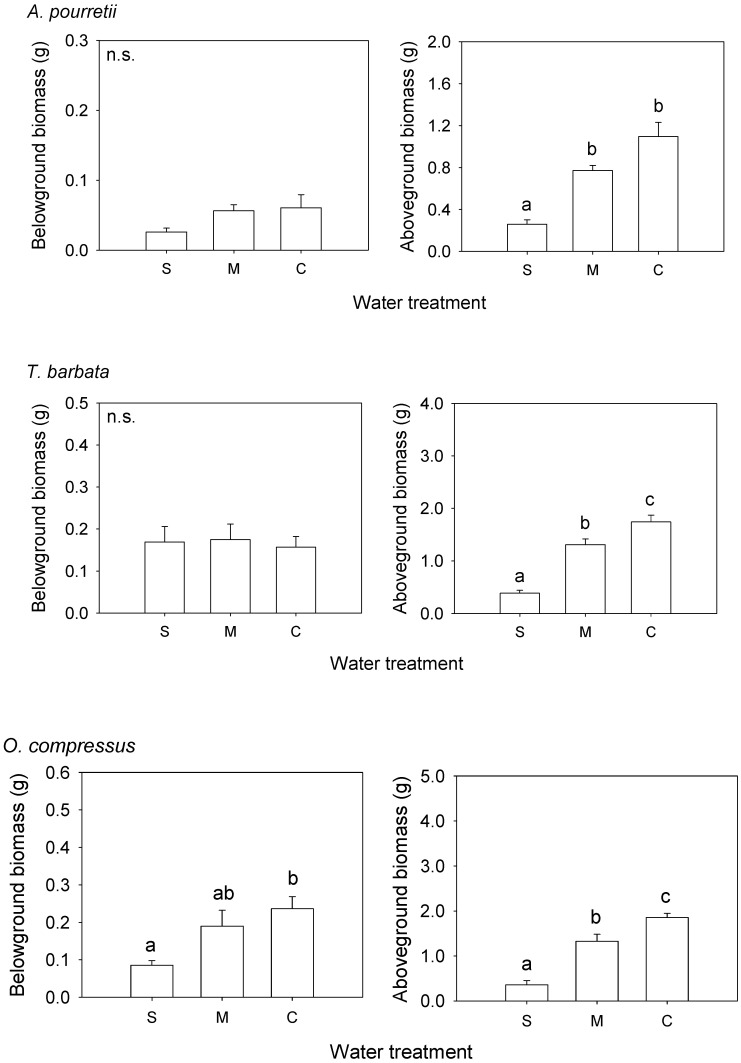

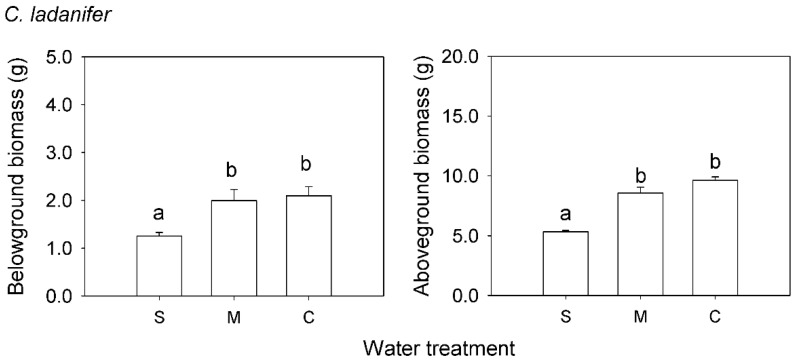
Belowground and aboveground biomass of *A. pourretti*, *T. barbata*, *O. compressus* and *C. salviifolius* in severe water deficit, moderate water deficit and control. Significant differences at *p* < 0.05 are indicated by different letters. n.s., not significant.

A common response of all herbaceous species to water deficit treatments in our study was the reduction in plant biomass production, but with an overall increase in root: shoot biomass. Aboveground growth is often more reduced than root growth due to water deficit, and an increase in root: shoot ratio is a common response that can be caused either by the increase in root growth or by a larger decrease in shoot growth compared to root growth [36,37]. In our study for *A. pourrettii* and *T. barbata*, this increase in root: shoot ratio was due to a decrease in above-ground biomass production, as belowground biomass did not differ among water treatments (Figure A1). 

The results are consistent with field data [2,32], showing that changes in water availability affect species functional groups differently, with legumes being the most affected, and moderate water deficit conditions seem not to impact plant physiology and morphology. The effects of both moderate and severe water deficit on *O. compressus* suggest that the presence of this species, and likely other water-sensitive legume species, may decrease in response to water deficit. On the contrary, *A. pourreti* and *T. barbata* have a higher ability to cope with water deficit, less affected by precipitation variability and/or drought events [2]. It has implications for plant community composition and diversity for forage production.

## 4. Materials and Methods

### 4.1. Experimental Set Up

Both the soil and plant species used in this experiment come from a Montado area in Southern Portugal. This Montado area is a Mediterranean evergreen oak woodland with mixed *Quercus suber* and *Quercus ilex* trees. The understorey vegetation consists of a mixture of C3 annual species, emerging after the first autumn rains and senescing late in spring, and shrubs, with *Cistus salviifolius* being the most common species. A total of 89 species were identified in the understorey at the Montado area, among which 19 grass (c. 40% plant cover), 41 forb (c. 38% plant cover), 17 legumes (c. 10% plant cover), and 1 shrub species (c. 9% plant cover). Despite a large number of species, the understorey is dominated by few species, with less than 10 species accounting for over 60% of plant cover. 

We selected four common Montado species based on our previous knowledge of local species and functional group composition: (1) the annual grass *Agrostis pourretii* Willd., one of the most abundant grasses at the study site (17% plant cover); (2) *Tolpis barbata* (L.) Gaertn., an endemic annual of the Asteraceae family and one of the most abundant forb species (6% plant cover); (3) *Ornithopus compressus* L., an annual legume commonly found at the study site (2% plant cover); (4) *Cistus salviifolius* L., a shrub belonging to the Cistaceae family (c. 9% plant cover).

The soil was sieved through a 5 mm mesh size and homogenized. The soil texture is sandy loam, and the field capacity is around 23%. The herbaceous plants were germinated in plug trays (in soil from the same site) and transferred into pots after four weeks. The pots (1.5 L) were filled with 1 L (c. 1.118 kg) of sieved fresh soil. One individual plant was planted into each center for six replicate pots per water treatment. For *Cistus salviifolius*, plants were collected in the field in December 2016, transported to the lab and placed into pots, as described above. The collected plants were selected to minimize variation in size, and plant size averaged 20 cm and ranged from 18 to 25 cm (average plant size per treatment: S = 21 cm; M = 20 cm; C = 19 cm). 

### 4.2. Water Deficit Treatment

The experiment was conducted in a climatic chamber at 16/8 h (day/night) photoperiod, and growth conditions (recorded using a datalogger) were: average temperature of 24 °C (ranging from 19 to 30 °C) and average air humidity of 50% (ranging from 35 to 65%).

The pots were randomly assigned to a position in the benches at the beginning of the experiment and rotated 1–2 times a week throughout the experimental time to account for microclimatic and light differences at different locations in the bench. Plants were kept over 80% of field capacity until the water deficit treatment application began, which started on 3 March 2017. Pots were subjected to three water treatments: (1) well-watered (control; C), where pots were kept over 80% of soil water capacity; (2) moderate water deficit (M), where pots were kept at 50–40% of soil water capacity; (3) severe water deficit (S), where pots were kept around 20% of soil water capacity. Six pots from each plant species were randomly assigned to each water treatment. Soil water content was maintained by weighing the pots every two days and rewetting them to the required water levels (80%, 50–40% or 20%).

### 4.3. Sampling and Measurements

About four weeks after the beginning of the water deficit treatment, photosynthesis was evaluated through the measurement of the light-dependent reactions of photosynthesis, chlorophyll, and fluorescence parameters, and leaves were sampled for physiological parameters and leaf dry matter content (LDMC). Afterwards, plants were harvested, above and belowground biomass was separated, and roots were carefully washed to eliminate attached soil particles. Aboveground biomass was also separated into reproductive and vegetative biomass. Vegetative, reproductive and root biomass was oven dried at 50 °C until constant weight. Leaf litter shed by *C. salviifolius* during the experiment was collected and oven dried at 50 °C until constant weight. Photosynthetic pigments and carbohydrates were analyzed on fresh leaves. Leaf dry matter content was determined following standard methodologies [38].

#### 4.3.1. Plant Water Content, Chlorophyll *a* Fluorescence and Pigments Content

Plant water content was determined through the leaf relative water content (RWC). Fresh leaves were harvested, and the fresh weight was determined. The leaves were placed in closed tubes filled with water and left overnight in dark and cold conditions to determine the turgid weight. Then, the leaves were dried at 80 °C until constant weight and the dry weight was determined. The RWC was calculated as RWC (%) = ((fresh weight − dry weight)/(turgid weight − fresh weight)) × 100.

Chlorophyll fluorescence was measured using the portable photosynthesis system LI-6400XT (LI-COR, Lincoln, NE, USA). After dark adaptation (for at least 30 min), the minimum fluorescence was measured by applying a weak intensity modulated light. The maximum fluorescence was measured after applying a saturating pulse of white light. Then, leaves were adapted to ambient light conditions, and after establishing a steady-state fluorescence, a saturating light was applied to determine the maximal fluorescence. Finally, after turning off the actinic light, minimal fluorescence was determined. The maximum quantum efficiency of photosystem II (F_v_/F_m_), the effective quantum efficiency of PSII (Φ_PSII_), the photochemical quenching (qP) and the non-photochemical quenching (NPQ) were calculated according to van Kooten and Snel [39].

Chlorophyll *a* (Chl *a*), chlorophyll *b* (Chl *b*) and carotenoids were quantified as described by Sims and Gamon [40]. To extract photosynthetic pigments, leaf discs were homogenized with acetone:50 mM Tris (80:20) in ice and dark conditions. After centrifugation (5000× *g* for 5 min at 4 °C), the absorbance of the acetone extracts was read at 470, 537, 647 and 663 nm in a microplate reader EnSpire (PerkinElmer). The contents of pigments were calculated according to Sims and Gamon [40].

#### 4.3.2. Plant Carbohydrates Contents

Total soluble sugars (TSS) were determined according to Irigoyen et al. (1992) with some modifications. First, leaf discs were homogenized with ethanol at 80% (*v*/*v*) and placed in a bath at 80 °C for one hour. After centrifugation (5000× *g* for 10 min at 4 °C), 30 µL of the supernatant was incubated for 10 min at 100 °C with an anthrone solution that contained 40 mg of anthrone, 1 mL of dH_2_O and 20 mL of H_2_SO_4_. After cooling and centrifugation (as described previously), the absorbance of the supernatant was read at 625 nm in a microplate reader EnSpire (PerkinElmer). TSS content was calculated using a glucose standard curve (y = 7.197x + 0.07, R^2^ = 0.985). 

For starch determination, leaf discs were homogenized with perchloric acid (30%, *v*/*v*) and incubated at 60 °C for one hour [41]. After centrifugation (10,000× *g* for 10 min at 4 °C), the supernatant was incubated with an anthrone solution (as described for TSS) at 100 °C for 10 min. Then, the samples were centrifuged (5000× *g*, 10 min, 4 °C), and the absorbance was read at 625 nm in a microplate reader EnSpire (PerkinElmer). Starch content was calculated using a glucose standard curve (y = 3.84x + 0.03, R_2_ = 0.992).

### 4.4. Statistical Analyses

To test for the effect of water deficit treatment on the measured parameters, a one-way ANOVA was performed. To access the magnitude of response of RWC to severe water deficit and moderate water deficit for each functional group, we calculated the response ratio (R, according to Hedges et al. [42] for every plant growing under water deficit as R = ln(Treatment/Control), where treatment refers to the RWC value obtained for the plant under water deficit. Control refers to the mean value of RWC obtained for the plants of each functional group under control conditions. Values closer to zero indicate no response of RWC to water deficit. Values significantly lower or higher than zero indicate a negative or positive response, respectively, of RWC to water deficit. One-sample *t*-test was used to test if the response ratio differed significantly from zero. The analyses were done using R version 3.3.2 [43] and TukeyHSD for Multiple comparisons after analysis of variance. Principal component analysis (PCA) was performed with CANOCO v4.02.

## 5. Conclusions

In conclusion, we characterized the physiological performance of four Mediterranean species to different levels of water deficit conditions and demonstrated that functional groups respond differently. The legume species *O. compressus* showed higher sensitivity to water deficit, reducing water status, particularly under severe conditions, and readjusting carbohydrate and pigment levels. In turn, *A. pourreti* and *T. barbata* showed a higher ability to cope with water deficit stress, maintaining water status. The shrub, *C. salviifolius*, despite the reduction in the RWC, seems better suited to handle water deficit stress conditions, possibly due to the activation of the stress-protective mechanism. These data will help to understand future plant community compositions, diversity, and forage production in the Mediterranean area, one of the most vulnerable to climate change.

## Figures and Tables

**Table 1:** Results from the one-way ANOVA testing for the effect of water treatment (severe water deficit, moderate water deficit and control) on the response of morphological and physiological traits measured. Significant *p* values (*p* < 0.05) are highlighted in bold.

Effect	Water Treatment											
Species	*A. pourretti*			*O. compressus*			*T. barbata*			*C. salviifolius*		
Response Variables	df	F	*p*	df	F	*p*	df	F	*p*	df	F	*p*
Physiological												
F_v_/F_m_	2	1.20	0.327	2	2.10	0.157	2	0.91	0.425	2	0.23	0.800
Φ_PSII_	2	0.44	0.653	2	0.55	0.589	2	**9.31**	**0.003**	2	1.28	0.306
NPQ	2	0.31	0.735	2	2.79	0.094	2	0.11	0.899	2	0.56	0.584
qP	2	0.41	0.671	2	0.09	0.919	2	0.15	0.859	2	0.61	0.559
Chl (*a* + *b*)	2	3.50	0.057	2	20.02	**<0.001**	2	0.97	0.404	2	1.43	0.275
Carotenoids	2	**5.30**	**0.018**	2	34.75	**<0.001**	2	1.46	0.265	2	1.24	0.321
Total Soluble Sugars	2	1.44	0.270	2	19.39	**<0.001**	2	**8.29**	**0.004**	2	0.79	0.471
Starch	2	0.93	0.417	2	19.25	**<0.001**	2	0.04	0.961	2	1.16	0.339
Morphological												
LDMC	2	**7.07**	**0.009**	2	48.09	**<0.001**	2	0.67	0.528	2	**15.66**	**<0.001**
Total biomass	2	**30.01**	**<0.001**	2	25.65	**<0.001**	2	**36.01**	**<0.001**	2	**62.99**	**<0.001**
Root: Shoot	2	**4.95**	**0.022**	2	12.62	**0.001**	2	**9.12**	**0.003**	2	0.27	0.764
Belowground biomass	2	2.34	0.131	2	6.02	**0.012**	2	0.13	0.877	2	**6.69**	**0.008**
Aboveground biomass	2	**32.72**	**<0.001**	2	39.1	**<0.001**	2	**38.01**	**<0.001**	2	**58.08**	**<0.001**

**Table 2:** Mean values (±SE) of the number of flowers (*O. compressus*) or inflorescences (*A. pourretti, T. barbata*) of plants grown under severe water deficit (S), moderate water deficit (M) and control (C) conditions and results from the one-way ANOVA testing for the effect of water treatment. Significant differences at *p* < 0.05 among treatments are indicated by different letters.

Species	Water Treatment			
	Severe Stress	Moderate Stress	Control	ANOVA Results
*A. pourretti*	6.17 ± 0.54 (a)	22.50 ± 2.69 (b)	28.33 ± 2.29 (b)	*F* = 30.97, *p* < 0.001
*O. compressus*	0.00 ± 0.00 (a)	15.17 ± 1.92 (b)	17.67 ± 2.62 (b)	*F* = 26.02, *p* < 0.001
*T. barbata*	10.80 ± 3.6 (a)	42.67 ± 4.5 (b)	47.67 ± 4.17 (b)	*F* = 21.41, *p* < 0.001

## Data Availability

Data not available in supplementary material is available from the authors upon request.
